# Multimodal prediction of the need of clozapine in treatment resistant schizophrenia; a pilot study in first-episode psychosis

**DOI:** 10.1016/j.bionps.2024.100102

**Published:** 2024-12

**Authors:** Jonatan M. Panula, Athanasios Gotsopoulos, Jussi Alho, Jaana Suvisaari, Maija Lindgren, Tuula Kieseppä, Tuukka T. Raij

**Affiliations:** a Department of Psychiatry, University of Helsinki and Helsinki University Hospital, Helsinki, Finland; b Department of Neuroscience and Biomedical Engineering, Aalto University School of Science, Espoo, Finland; c Advanced Magnetic Imaging Center, Aalto University School of Science, Espoo, Finland; d Mental Health, Public Health and Welfare, Finnish Institute for Health and Welfare, Helsinki, Finland

**Keywords:** Treatment resistant schizophrenia, First-episode psychosis, Machine learning, Neural networks, Inter-subject correlation analysis

## Abstract

As many as one third of the patients diagnosed with schizophrenia do not respond to first-line antipsychotic medication. This group may benefit from the atypical antipsychotic medication clozapine, but initiation of treatment is often delayed, which may worsen prognosis. Predicting which patients do not respond to traditional antipsychotic medication at the onset of symptoms would provide fast-tracked treatment for this group of patients. We collected data from patient records of 38 first-episode psychosis patients, of whom seven did not respond to traditional antipsychotic medications. We used clinical data including medical records, voxel-based morphometry MRI data and inter-subject correlation fMRI data, obtained during movie viewing, to predict future treatment resistance. Using a neural network model, we correctly predicted future treatment resistance in six of the seven treatment resistance patients and 25 of 31 patients who did not require clozapine treatment. Prediction improved significantly when using imaging data in tandem with clinical data. The accuracy of the neural network model was significantly higher than the accuracy of a support vector machine algorithm. These results support the notion that treatment resistant schizophrenia could represent a separate entity of psychotic disorders, characterized by morphological and functional changes in the brain which could represent biomarkers detectable at early onset of symptoms.

## 1 Introduction

Despite rigorous work on treatment options, and more than 50 antipsychotic medications developed since the discovery of chlorpromazine in 1952, few major breakthroughs on enhancing the long-term outcome of schizophrenia have been made in the past decades (Hegarty et al., 1994, Jaaskelainen et al., 2013). For a substantial number of patients (as high as 30 %), whose symptoms respond inadequately to traditional antipsychotic medications, clozapine can provide superior symptom alleviation (Brenner et al., 1990, Gillespie et al., 2017, Kane et al., 1988, Siskind et al., 2022). However, the risk for severe side-effects, including agranulocytosis, limits its use to treatment-resistance cases who commit to strict laboratory follow-up. The current Finnish guidelines define treatment-resistance as a failure to achieve symptom alleviation after treatment attempts of six weeks using two different first- or second generation antipsychotic medications (Schizophrenia: Current Care Guidelines, 2022). Establishing treatment resistance and initiating clozapine treatment in lieu of other antipsychotic medication in patients is often delayed, leading to prolonged symptomatic illness, and possibly predisposing for less favorable outcomes (Stokes et al., 2020).

Clinical variables such as male sex, young age at onset of symptoms, long duration of untreated psychosis, medical non-adherence and social disadvantage, have been associated with the development of treatment resistant schizophrenia (TRS) in first-episode psychosis (FEP) patients (Smart et al., 2022, Suvisaari et al., 2018). Such mostly univariate associations have failed to lead to working prediction models applicable in clinical practice (Dwyer et al., 2018). Although recent work using larger cohorts and multivariate analyses shows promising results (area under curve 0.59 – 0.78), more reliable models are needed to change current guidelines on clozapine treatment initiation (Ajnakina et al., 2020, Barone et al., 2022, Osimo et al., 2023, Smart et al., 2022, Wong et al., 2024). In a recent study, Yang et al. showed increasing accuracy when predicting TRS in an FEP cohort when combining clinical data with selected structural MRI variables and glutathione levels in the anterior cingulate cortex, suggesting that a multimodal approach could provide superior accuracy (Yang et al., 2022). Supporting a multimodal approach, Koutsouleris et al. showed a significant increase in performance when imaging data was combined with clinical data to predict functional outcomes in clinical high-risk (CHR) state for psychosis patients (Koutsouleris et al., 2018, Koutsouleris et al., 2021). Even though resting-state fMRI changes have been documented in TRS (Chan et al., 2019), it is not known whether they are present at the onset of symptoms and could improve on the prediction accuracy provided by clinical variables and structural MRI data. It has, however, been suggested that TRS prediction could be a key target for fMRI in clinical applications (Voineskos et al., 2024).

Deep learning algorithms, including neural networks (NN), have emerged as the standard method in fields such as speech recognition and computer vision, but only recently gained ground in classification tasks associated with brain disorders, and show promising results when compared to more traditional algorithms such as support vector machines (SVM) (Bernal et al., 2019, Iyortsuun et al., 2023, Meszlenyi et al., 2017, Pinaya et al., 2016). We elected to include the SVM as a baseline comparison to evaluate the performance of the NN. To our knowledge, there is no study directly comparing SVM and NN accuracy in predicting TRS based on brain imaging. A deep learning approach has, however, outperformed an SVM algorithm when classifying patients with schizophrenia and control subjects using structural MRI data (Pinaya et al., 2016). While NN-algorithms have not been employed as commonly in psychiatric disorders, they are predicted to possibly offer a more individualistic assessment of diagnosis and clinical outcome (Durstewitz et al., 2019, Vieira et al., 2017).

This study had three goals: First, to examine whether it is possible to predict treatment-resistance and subsequent need for clozapine use in FEP patients. Second, to study whether we could improve on the accuracy of said prediction by including multiple modalities in the algorithm; and finally, to compare the performance of an NN algorithm to the more traditionally used SVM algorithm.

## 2 Methods

### 2.1 Participants

The subjects included in this research were selected from the Helsinki Early Psychosis Study (HEPS) (Mantyla et al., 2015), which includes FEP patients recruited from psychiatric clinics and wards within the Hospital District of Helsinki, of whom 97 participated in imaging studies. Symptom scores and diagnoses were verified by a senior psychiatrist (J.S). Positive symptoms were rated using the Brief Psychiatric Rating Scale Extended version (BPRS-E) (Ventura et al., 1993). Psychosis was defined as a score of four or higher for either hallucinations (BPRS-E item 10) or unusual thought content (BPRS-E item 11), which were also the inclusion criteria for patients in the study. In addition, patients were assessed using the The Scale for the Assessment of Negative Symptoms (SANS), Global Assessment of Functioning (GAF), Social and Occupational Functioning Assessment Scale (SOFAS), Mood Disorder Questionnaire (MDQ), Alcohol Use Disorders Identification Test (AUDIT), Beck Depression Inventory (BDI), Beck Anxiety Inventory (BAI), and Obsessive-Compulsive Inventory (OCI-R). Furthermore, the clinical variables contained estimates of duration of untreated psychosis, body mass index (BMI), high-sensitivity C-reactive protein (S-hs-CRP) levels, information on education, occupational status, adverse events in childhood, and substance abuse (Supplement 1). Before participating in the study, all patients submitted a written consent. The HEPS project has been approved by the Ethics Committee of the University Hospital District of Helsinki and Uusimaa.

**Fig. 1:**
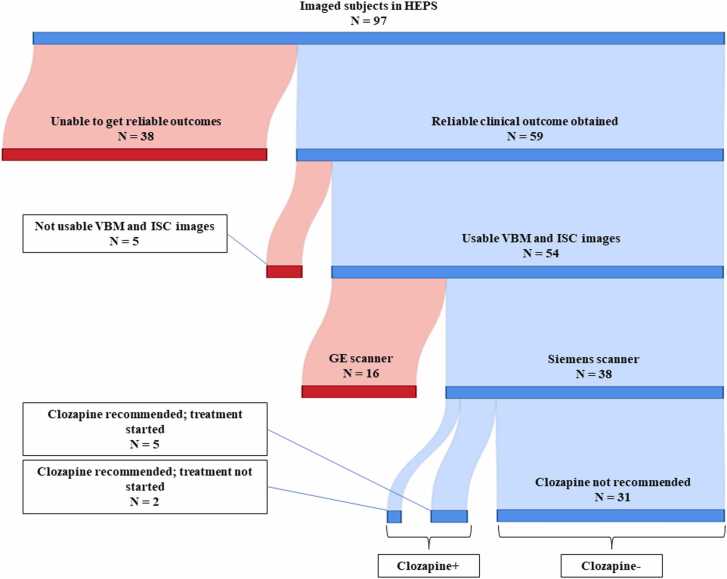
Sankey diagram of patient selection. HEPS = Helsinki Early Psychosis Study.

In lieu of the more traditional approach of using clozapine prescriptions as an indication of treatment resistance, we elected to gather information about indication for clozapine from patient records, as there are multiple reasons why patients would not be prescribed said medicine even though there would be adequate indications to do so. We excluded patients who did not give their consent for studying their medical records, had moved to other cities or other areas in the municipality that use different electronic patient record systems, and patients who dropped out of psychiatric care, committed suicide during the follow-up or patients whose images could not be included in the study (Fig. 1). After exclusion, we obtained sufficient information for 38 FEP patients, of whom seven were recommended clozapine. As the outcome in our study was defined as an indication of clozapine treatment, we named the groups as the clozapine+ group and clozapine- group. For a detailed description of patient selection see Fig. 1.

### 2.2 MRI acquisition

During fMRI scanning, FEP patients viewed scenes from the movie “Alice in Wonderland” (Tim Burton, Walt Disney Pictures, 2010; Finnish soundtrack. License to present the movie was purchased from the local right holder M&M Viihdepalvelut Inc.) for a duration of 7 minutes and 21 seconds. For a detailed review of the scenes please see (Rikandi et al., 2017). In short, the movie starts at a garden party, where the main character Alice sees a rabbit whom she follows to the roots of a tree. Leaning over, she falls into a deep hole, where she sees levitating objects on her way down. She crashes through the roof of a round room, where she grows tremendously after eating a piece of cake and shrinks after drinking a potion. Thenceforth, she walks into Wonderland, where she meets multiple surreal characters. Finally, she walks through a dark forest, where she encounters the levitating Cheshire cat. The scenes were selected to contain both realistic and unrealistic content, hypothesizing that unrealistic scenes might evoke different responses in psychotic subjects when compared to realistic content. In overlapping samples, the content was rated as more unrealistic by control subjects when compared to FEP patients (Panula et al., 2023), and abnormal activation of the salience network in delusional FEP patients was mostly driven by the scenes that an independent reference group evaluated as more unrealistic (Panula et al., 2022).

MRI images for the patients included in this study were obtained with a Siemens Skyra 3-T scanner at Aalto University, Espoo, Finland. Blood oxygen level dependent (BOLD) signal was acquired with gradient echo-planar imaging (EPI) sequence (TR 1.8 seconds, echo time 30 ms, flip angle 75°, FOV 240 mm). T1 structural images were obtained with MPRAGE-sequence (TR/TE 2530 ms/3.3 ms, FOV 256 mm, slice thickness 1 mm, voxel size 1 ×1×1 mm³, number of slices 176).

### 2.3 MRI pre-processing

We pre-processed structural MRI data by using the SPM12 software (http://store.elsevier.com/product.jsp?isbn=9780123725608). We extracted, realigned, segmented, and normalized the images to the Montreal Neurological Institute (MNI) template using the DARTEL-toolbox. We resliced voxels to a size of 2 ×2×2 mm and smoothed the images with a gaussian kernel of 6 mm full width at half maximum (FWHM). We used unmodulated voxel-based morphometry (VBM) images to analyse changes in grey matter concentration (Ashburner and Friston, 2000, Good et al., 2001).

fMRI data was pre-processed using the FSL software (www.fmrib.ox.ac.uk) and a custom-made MATLAB (MathWorks Inc., Natick, MA, USA) code (BRAMILA pipeline v2.0, available at https://version.aalto.fi/gitlab/BML/bramila). We corrected EPI images for slice timing differences and head motion, and co-registered them to the participant’s structural image. Finally, we normalized the images to the MNI-template. We smoothed the functional images with a gaussian kernel of 6 mm FWHM. To account for scanner drift, we applied a Savitzky-Golay filter (Cukur et al., 2013) and a high-pass temporal filter. To account for motion and physiological artefacts, we modified the BOLD-signal using 24 motion-related regressors in addition to signals from cerebrospinal fluid and white matter (Power et al., 2012). Following this, we calculated inter-subject correlations (ISC) (Hasson et al., 2004) using an independent reference group of 44 age- and sex-matched control subjects chosen from the civil registry, who viewed the same movie scenes as the FEP patients. For each subject, we obtained a single ISC brain map by calculating all subject-pairwise Pearson’s correlation coefficients of the voxel-time-courses with respect to the independent reference group and then averaging them after the Fisher’s Z-transformation.

### 2.4 Data preparation

The clinical data consisted of 81 pre-determined clinical variables (for selected variables of both groups see Table 1 and for all variables see Supplement 1). Some variables contained random missing values. We imputed missing values after randomly splitting the subjects into training and testing groups. Missing values were imputed in both the training and testing datasets using values obtained from the training data (mean value for scalar/ordinal variables and mode/unknown for binary and categorical variables). The only exception was Fagerstrom Test for Nicotine Dependence, which was imputed as 0 for non-smokers. Finally, we normalized the clinical variables to values between 0 and 1.

**Table 1:** Demographic information.

	Clozapine+^a^N=7	Clozapine-aN=31	Group Difference^b^
Male	6 (85.7), (0)	19 (61.3), (0)	p = 0.238
Age	25.1, 25.2 (22.4–28.0, 5.1), (0)	26.8, 25.8 (18.3–39.1, 9.9), (0)	p = 0.883
DUP	1=3, 2=2, 4=2, (0)	1=14, 2=11, 3=2, 4=3, (1)	p = 0.662
Currently smoking	3 (42.9), (1)	5 (15.6), (3)	p = 0.082
Nicotine dependence scale	4.3, 5.0 (0−8, 8), (1)	1.5, 0.0 (0−7, 3), (5)	p = 0.098
Cannabis ever used	5 (71.4), (0)	15 (48.4), (0)	p = 0.270
S-hs-CRP	4.2, 1.7 (0.2–12.5, 10.0), (0)	1.0, 0.4 (0.1–4.3, 1.5), (3)	p = 0.066
GAF	32.0, 35.0 (15−40, 3), (0)	39.4, 40.0 (30−65, 5), (0)	**p = 0.027**
Audit	11.8, 8.5 (2−30, 16), (1)	7.1, 6.8 (0−21, 12), (4)	p = 0.260
BPRS-E scale at admission			
Item 10 Hallucinations	4.1, 5.0 (1−6, 4), (0)	2.9, 2.0 (1−6, 4), (0)	p = 0.145
Item 11 Unusual thought content	5.3, 6.0 (1−7, 1), (0)	3.7, 4.0 (1−7, 4), (0)	**p = 0.015**
Item 12 Bizarre behavior	1.4, 1.0 (1−2, 0), (0)	1.5, 1.0 (1−6, 1), (0)	p = 0.606
Item 15 Conceptual disorganization	1.0, 1.0 (1−1, 0), (0)	1.2, 1.0 (1−3, 0), (0)	p = 0.530
Item 16 Blunted affect	2.1, 1.0 (1−5, 2), (0)	1.6, 1.0 (1−4, 1), (0)	p = 0.530
SANS scale at admission			
Alogia	0.1, 0.0 (0−1, 0), (0)	0.3, 0.0 (0−3, 0), (0)	p = 0.999
Anhedonia	1.4, 1.0 (0−3, 1), (0)	1.5, 2.0 (0−4, 3), (0)	p = 0.999
Avolition-apathy	3.0, 3.0 (2−4, 0), (0)	1.8, 2.0 (0−3, 2), (0)	**p = 0.019**
Diagnosis at 1-year follow-up	295.3 Paranoid schizophrenia (n=2) 295.7 Schizoaffective disorder (n=1) 295.9 Schizophrenia undefined (n=4)	295.3 Paranoid schizophrenia (n=3) 295.4 Schizophreniform disorder (n=7) 295.9 Schizophrenia undefined (n=2) 296.04/296.44 Bipolar type 1 disorder (n=6) 296.24/296.34 Major depressive affective disorder with psychotic features (n=3) 298.8 Brief psychotic disorder with psychotic features (n=3) 298.9 Unspecified psychosis (n=7)	

DUP = duration of psychosis (1 = < 1 month, 2 = 1 – 6 months, 3 = 6 – 12 months, 4 = > 12 months), S-hs-CRP = high-sensitivity C-reactive protein, GAF = Global assessment of functioning, Audit = Alcohol Use Disorders Identification Test, BPRS-E = Brief Psychiatric Rating Scale, SANS = Scale for the Assessment of Negative Symptoms

a Frequency (%) or mean, median (range, IQR), (missing values N)

b Mann-Whitney U-test or Pearson Chi-square test. Significant uncorrected p-values shown in bold.

For comparison, we used a univariate statistical test on the variables between the two groups, comprising a Mann-Whitney U-test for scalar variables and a Pearson Chi-square test for categorical variables. P-values were adjusted for multiple comparisons using the Bonferroni correction.

**Fig. 2:**
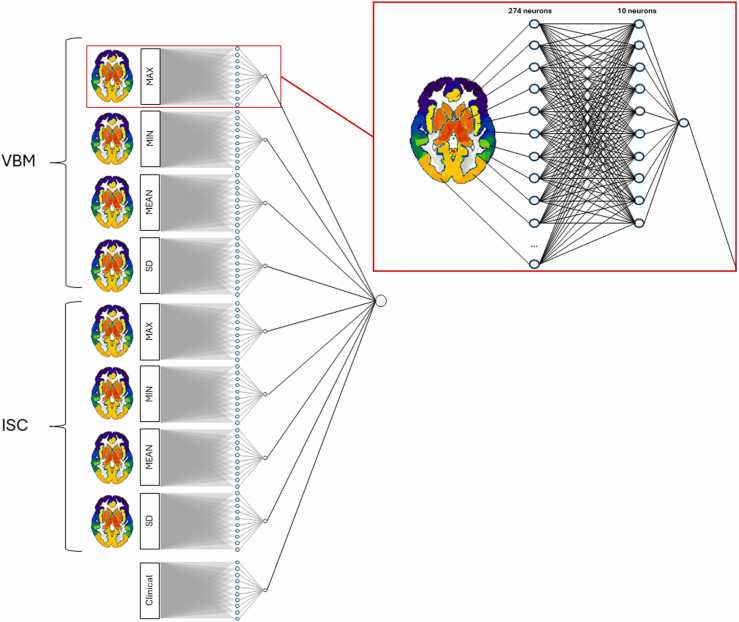
Structure of the neural network. The network combines one clinical modality and a total of eight image measures created from VBM and ISC images. The clinical data contains 81 variables normalized to values between 0 and 1. Missing values were imputed using the mean or mode of the training dataset. The VBM and ISC images were segmented using the Brainnetome atlas into 274 regions of interest (ROI), which were converted into four matrixes using min-, max-, mean-, and SD-pooling from each ROI of the atlas. The clinical data contained 81 initial nodes, and each of the eight imaging maps contained 274 initial nodes (corresponding to the number of parcellations in the atlas). Each initial node in each of the nine networks was connected to ten nodes in the first hidden layer, and these ten nodes were connected to one node in the second hidden layer. The nine nodes in the second hidden layer were connected to a common output node. Brain image created with the MRIcroGL software (Rorden and Brett, 2000).

Instead of making prior assumptions on which brain areas to include in the analysis, we elected to use segmented whole-brain data. Due to the computational demand of permutation analyses, we used region-wise pooling to reduce the number of variables in the MRI data. We separately segmented both image modalities (VBM and ISC) using the Brainnetome atlas (Fan et al., 2016), and computed minimum, maximum, mean and standard deviation (SD) values of the voxels within each region in the atlas (274 regions). Thus, each region in the Brainnetome atlas was represented in eight segmented maps for a total of 2192 MRI variables (Fig. 2).

### 2.5 Predictive algorithms

For the machine learning algorithms, we prepared and analyzed the data using an in-house developed software based on MATLAB (https://github.com/gostopa1/DeepNNs), allowing for the selection of any number of clinical and/or image-modalities including structural and functional MRI-data. For this study, we used clinical data, VBM data and ISC data.

The software used for analyses contains parallel SVM- and NN-algorithms. For the SVM algorithm, we used the built-in function in MATLAB, using the default linear kernel for binary classification. The topology of the neural network with two hidden layers and nine parallel subnetworks is visualized in Fig. 2. To allow for different data patterns to be learned, in the parallel networks, each of the 81 clinical variables and 274 values of each of the eight brain image maps connect to ten unique hidden nodes in the first layer, and these ten nodes connect to a single node in the next layer. The hidden nodes in the second hidden layer connect to a common output node. We elected to train each subnetwork specifically for the clinical data and each pooled brain image map and omitted connections between these before connecting the second hidden layer to the common output node. The reasoning behind the proposed sparse architecture of the neural network is threefold; First, the sparsity reduces training speed as only non-zero weights are optimized; Second, our design does not allow for connections across multiple modalities before the last layer. As such, the inequal number of features per modality does not introduce any bias to the model. Third, the multiple neurons in the first hidden layer allow for learning distinct features per modality while the output layer allows for direct interpretation of the contribution of each modality. We elected to use a randomized pair cross validation without resampling, with one randomly selected subject from each group as the test data (Shan, 2022). We retrained the network 2000 times with randomized subject pairs for each run. Subjects from the larger group were randomly omitted to create groups of equal size in the training data for each run to avoid favoring one class over the other due to unequal sample size. Each run was trained for 10 000 iterations, with a batch size of 10 and a learning rate of 0.0001 using ADAM as the optimization algorithm. Every node in the network used a sigmoid/logistic activation function.

We measured the accuracy of both algorithms using two different methods. First, we obtained the predicted class of the two subjects in the testing dataset for each run and compared them to the true labels. Thus, the accuracy for each run was either 0 %, 50 % or 100 %. The final accuracy was calculated as the average accuracy over the 2000 runs. Second, we assessed accuracy using ensemble hard voting across the 2000 runs to predict a final class for each subject based on the more prevalent prediction across all runs and compared this voted label to the true label for each patient. We calculated the significance of each prediction by permuting the labels and calculating 1000 permutation analyses with shuffled labels. We calculated accuracies for the clinical data, VBM data and ISC data separately, and for all possible pairwise combinations before finally combining all the three modalities together. For the average accuracy, we calculated 95 % confidence intervals using bootstrapping with 10 000 resampling runs.

To calculate whether the three combined modalities provided significantly greater accuracies than any single or pairwise combination of modalities, or compare the two algorithms to each other, we used a non-parametric two-sample permutation test with 1000 permutations. Each analysis contained 2000 individual runs with randomly selected test pairs, with an accuracy of 0 %, 50 % or 100 %. When comparing two analyses, the absolute mean difference in accuracy between the two analyses was compared to the distribution of absolute mean accuracy difference between the two permuted groups obtained by randomly shuffling the accuracies of individual runs between the two groups. The lowest possible obtainable two-sided p-value was 0.000999…, in the case where the absolute mean accuracy difference between the two analyses was larger than any of the 1000 permutation tests.

While the prediction accuracies of single modalities (clinical, VBM or ISC) could reflect the relative contribution to the multimodal prediction, we attempted to validate these results by comparing the contribution of each modality to the final accuracy by analyzing the importance values calculated using Layerwise Relevance Propagation (LRP) as suggested by Montavon et al. (Montavon et al., 2017). The importance values were calculated by redistributing the output of the classifier back to the inputs, assessing importance values that correspond to the contribution of each variable to each class. Each run of the NN algorithm attempts to predict the class for two subjects, one from each class. We combined the importance values for each variable in predicting each subject to the correct class, regardless of whether the subject was correctly predicted or not in said run. The importance values for each variable were summed over the 2000 runs. We obtained importance values for each clinical value, and a total of 8 importance values for each brain region, representing the values from the minimum, maximum, mean and SD maps in VBM and ISC analyses. To obtain an estimate for the relative contribution of each of these eight brain maps to the final prediction, we calculated the mean importance value for the 274 regions in each map. In addition, to provide an estimate for which variables most significantly contributed to correct classification, in each of the brain maps and the clinical data, we ranked the features according to importance value for predicting the subjects in each class correctly and selected the 10 most important features in each. To visualize the results, we pooled results within the four ISC maps and the four VBM maps to obtain areas that at least in one of the four maps were among the 10 most significant areas in addition to the 10 most significant clinical variables.

## 3 Results

Of the 38 patients included in this study, seven were considered TRS, and recommended clozapine treatment. On average, clozapine treatment was suggested 497 days after the first contact with healthcare for psychotic symptoms. Across the 81 clinical variables, the two groups did not differ regarding age, sex, duration of psychosis or education. In addition, the groups did not differ regarding chlorpromazine equivalent doses during the baseline evaluation (p = 0.740). The proportion of missing values was 4.2 %, spread across 27 of the clinical variables. At an uncorrected p < 0.05, the clozapine+ group displayed higher scores on the BPRS-E item 11 (unusual thought content) during the week before the baseline interview, a lower GAF score, a higher score for SANS avolition and a lower score for amount of exercise (see Table 1 for selected results and Supplement 1 for full results). None of these results remained statistically significant after Bonferroni correcting for multiple comparison.

**Fig. 3:**
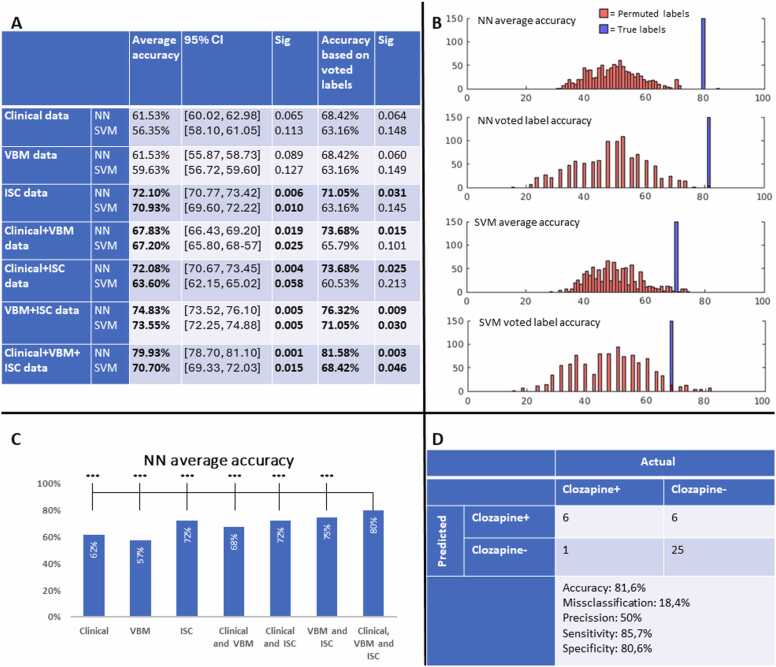
Summary of performance of NN and SVM algorithms in predicting need of clozapine use in first episode psychosis patients. **A**) Accuracies for SVM and NN predictive algorithms when using single modalities, pairwise combinations of modalities and clinical data, VBM data and ISC data combined. Significance calculated based on permutation tests with 1000 random permutation runs. Results significant at p < 0.05 are shown in bold. **B**) Visualized results of true label run and permutation runs with shuffled labels. Accuracy on X-axis, and number of permuted analyses on Y-axis. “True run” pilar enlarged for better visualization. **C**) Comparison of average accuracies in predicting need of clozapine using clinical data, VBM data and ISC data as single modalities, pairwise combinations or all three modalities together. All comparisons are visualized as two-sided p-values made in reference to the analysis with all modalities included. **D**) Confusion matrix displaying accuracy of NN algorithm based on voted label. VBM = voxel-based morphometry, ISC = inter-subject correlation, NN = neural network, SVM = support vector machine. *=p<0.05, **=p<0.01, ***=p<0.001, NS = not significant.

We predicted the clinical outcome using the two machine learning algorithms and two different methods for assessing accuracy. Accuracy was measured using mean accuracy of the pairwise prediction of subjects across the 2000 runs, as well as by voting a final class for each subject. Results for both methods, using single modalities or combined modalities, are presented in Fig. 3. When analyzing single modalities using either algorithm, only the ISC data provided significant mean accuracy. Isolated clinical data and VBM data provided non-significant, trend-like results close to 50 %. Using the voted label method, the results were similar, but the accuracy of the SVM model when using isolated ISC data was lower and non-significant.

In pairwise combinations of modalities, the highest mean accuracy for both algorithms was reached by combining VBM and ISC data, while the combination of clinical and ISC data provided the highest voted label accuracy using the NN algorithm. The performance of the NN algorithm was further enhanced by combining clinical data and both imaging modalities. The mean accuracy across the 2000 runs using all three modalities was 79.93 %, p = 0.001, 95 % CI [78.70, 81.10]. Using ensemble voting to predict a final class for each subject, the model reached an accuracy of 81.58 %, p = 0.003 Thus, the NN algorithm correctly predicted a future treatment failure in six of the seven patients of the clozapine+ group. Statistical significance, assessed by comparing the true run to permuted label runs, was observed for both algorithms with all three modalities analyzed in tandem. While the accuracy of the NN-algorithm increased as more modalities were combined, the accuracy for the SVM was highest when combining VBM and ISC data but decreased when clinical data was further combined.

To assess whether the accuracy reached by combining all three modalities was statistically superior to that of isolated or pairwise combination of modalities, we compared the absolute difference between mean accuracies to the difference between mean accuracies of permuted runs. When analyzed by the NN-algorithm, the mean accuracy with all three modalities was significantly higher than with any combination of two modalities. When comparing these results to the SVM model, the accuracy of the NN algorithm was significantly higher (p < 0.001).

**Fig. 4:**
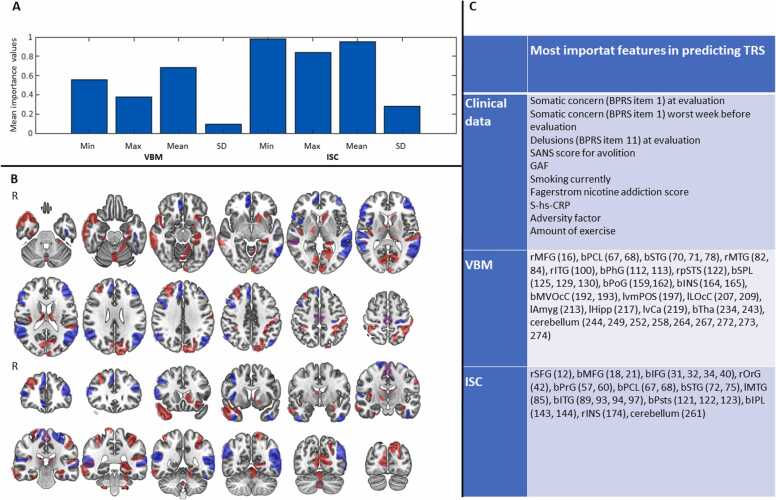
Visualization of importance results. **A)** Summed importance values for VBM and ISC minimum, maximum, mean and SD maps. **B)** Visualization of brain regions that were present among the ten most important areas in at least one of the minimum, maximum, mean or SD maps in VBM (red) and ISC (blue) analyses. **C)** Table with details (name and Brainnetome parcellation number for VBM and ISC) on most significant features in all modalities (l in abbreviation = left side of brain, r in abbreviation = right side of brain, b in abbreviation = bilateral). SFG = superior frontal gyrus, MFG = medial frontal gyrus, IFG = inferior frontal gyrus, OrG = orbital gyrus, PrG precentral gyrus, PCL = paracentral lobule, STG = superior temporal gyrus, MTG middle temporal gyrus, ITG = inferior temporal gyrus, PhG = parahippocampal gyrus, Psts = posterior superior temporal sulcus, SPL = superior parietal lobule, IPL = inferior parietal lobule, PoG = postcenral gyrus, INS = insular gyrus, MVOcC = medioventral occipital cortex, LOcC = lateral occipital cortex, Amyg = amygdala, Hipp = hippocampus, vCA = ventral caudate, Tha = thalamus. VBM = voxel-based morphometry, ISC = inter-subject correlation, vmPOS = ventromedial parietooccipital sulcus. Images shown in radiological convention. Brain image created with the MRIcroGL software (Rorden and Brett, 2000).

The most significant clinical variables and brain regions for predicting treatment resistance are presented in Fig. 4. In the analysis including all modalities, based on the summed importance values averaged within each MRI brain map, the SD maps seemed to be the least important for predicting patients to the correct class. On average, VBM importance values were lower than ISC importance values, mimicking the results from analyses on modalities in isolation.

## 4 Discussion

Our study investigated the prediction of future TRS in an FEP patient cohort employing a multimodal approach using clinical data in tandem with structural and functional MRI data. In addition, we compared the performance of an NN-algorithm and an SVM-algorithm using single and multiple modalities. Using a multivariate classifier, the future need for clozapine treatment was correctly predicted in six of the seven TRS patients in the FEP patient cohort. Increasing number of modalities enhanced accuracy, and the NN outperformed the SVM when all modalities were included. Using clinical data alone, our model did not reach statistical significance even though there was a trend like result using the NN algorithm (68.42 %, p = 0.064). In line with a similar study, these results improved when VBM data was added to the clinical variables (Yang et al., 2022). The most accurate isolated modality was the ISC data, and the accuracy of the model was significantly improved when this modality was combined with clinical data and VBM data (81.58 %, p = 0.003).

FEP cohorts constitute heterogeneous groups of patients, with widely varying outcomes ranging from complete remission to severe, lifelong disability (Lally et al., 2017). Although clozapine use can predispose to severe side-effects, and most FEP patients respond adequately to traditional and second-generation antipsychotic medication, we should acknowledge that the patients having TRS would most likely greatly benefit from earlier clozapine intervention. In general, all FEP patients are recommended to initiate antipsychotic medications at the beginning of treatment. Following current guidelines, patients presenting with symptoms fulfilling the criteria for schizophrenia and not-responding to first-line antipsychotic medications should be considered as TRS and evaluated for clozapine treatment. As delays in treatment initiation remain an issue, clinical decision support systems (CDSS) could provide a suitable aid to minimize this delay. The functionality of classification algorithms suffers when the difference between groups is ambiguous, as can often be the case in descriptive diagnostics in psychiatry. Much effort has been invested in classifying patients with schizophrenia and control subjects (Cortes-Briones et al., 2022). While many studies show high accuracies it is unclear if any of the changes observed in chronic schizophrenia translate to biomarkers when predicting outcomes in FEP patients. In addition, the value of classification of diagnoses or diagnostic subgroups remains questionable, as the impact on treatment based on diagnosis is miniscule (Hyman, 2010, Walter et al., 2019, Woo et al., 2017). Thus, developing classification algorithms directly for treatment selection might be more fruitful than common attempts to classify psychiatric diagnoses. The need for clozapine treatment provides a potential target for such algorithms, as the guidelines for clozapine initiations, at least in theory, are clear. While there is currently no consensus as to how to define TRS in predictive studies (Howes et al., 2017), many studies use clozapine prescription as a proxy for TRS (Osimo et al., 2023, Smart et al., 2021). While this information is most likely to represent most of these patients, there are still instances where treatment is not initiated despite patients fulfilling criteria for TRS. Such reasons include co-morbidities, low probability of treatment and follow-up adherence, or patients deciding not to use the medication for various reasons. To increase the validity of our results, we instead elected to gather this information from patient records. In our sample, two of the seven treatment resistant patients would have been misclassified had we elected to use prescription data as the outcome. Further, many studies compare TRS to treatment responsive schizophrenia instead of a more heterogenous population of FEP patients. Such more heterogenous cohorts naturally lead to a lower proportion of TRS (Siskind et al., 2022), as was the case in this study (18.4 %). We elected to study a FEP patient cohort to better represent the heterogenous disorders presenting with acute psychosis in the early stages of different disorders.

Despite the promisingly high sensitivity (85.7 %) and specificity (80.6 %) of our model, the low prevalence of TRS and uneven size of groups resulted in as many patients correctly predicted as future TRS to be misclassified as TRS (i.e., 50 % precision). Such bias is characteristic of models predicting rare syndromes. The risk of severe side effects of clozapine limits its use to most severe cases, and even if less severe cases might respond to clozapine as well, they should instead use safer second generation anti-psychotic medication. The most likely goal of a CDSS would be to fast-track initiation of clozapine in FEP patients classified as future TRS, and a low specificity would lead to initiation of clozapine in patients who would respond to safer medication. Large clinical cohorts have now been studied, and while the results are promising, they still lack the sensitivity, and especially specificity, needed for clinical practice. Using imaging data in tandem with clinical data is a promising prospect, but larger multicenter studies are still needed. Genome-wide association studies (GWAS) provide another interesting potential predictor for TRS. More recent GWAS benefit from extremely large cohorts, increasing the robustness of the results and providing a possible foundation for a CDSS, but as of yet, no study has provided high enough accuracies to be considered as a candidate (Pisanu and Squassina, 2019). More sophisticated pattern recognition models, spanning multiple modalities, in tandem with larger cohorts could pave the way for prognostic models and individualized care in psychiatry (Griffiths and Birchwood, 2020, Korda et al., 2021). Combining GWAS or EEG/MEG data with imaging data could provide higher accuracies than either type of data analyzed separately.

While the goal of predictive algorithms in clinical settings is to improve care and diagnostics, they can additionally provide insight into underlying mechanisms, which could indirectly benefit patients through development of new treatments. Due to the nature of predictive algorithms, it is, however, difficult to draw direct conclusions about the relationship between predictors and outcomes. To highlight relevant areas, we extracted individual importance values based on weights in the NN algorithm (Fig. 4). These results should, however, be approached with reservations as the multivariate relationship between variables is lost in such analyses. Nevertheless, many of these regions, such as the insula, caudate, amygdala, hippocampus, thalamus, and the parietal and temporal cortex, have been associated with TRS (Barry et al., 2019, Liu et al., 2022). While the clinical data itself did not provide significantly better accuracy than chance, adding this data to the imaging data significantly improved the accuracy. A majority of the 10 most important clinical variables were significant or close to at an uncorrected p-value of < 0.05 when comparing the two groups (Supplement 1). The clozapine+ group displayed significantly higher scores for SANS avolition, a similar result as observed by Yang et al. in their model for predicting TRS in an FEP cohort (Yang et al., 2022). The clozapine+ group had more severe delusions when evaluated at baseline. Similar results, where more severe positive symptoms predict TRS when compared to treatment responders have been observed in a retrospective study (Barone et al., 2022). In addition to more severe positive and negative symptoms, a lower level of functioning, as measured by GAF score, was also predictive of TRS. Smoking was more prevalent in the clozapine+ group, their scores for nicotine addiction were higher, and they displayed higher S-hs-CRP values. Biomarkers of inflammation, such as a higher CRP level, have been associated with the development of TRS (Jiao et al., 2022), and more prevalent smoking could be associated with oxidative stress. Smoking has previously been shown to be more prevalent in TRS (Kennedy et al., 2014), but it is unsure whether there is a causal relationship between oxidative stress and more severe psychotic disorders (Buosi et al., 2021). More frequent childhood adversities were predictive of TRS. Prevalent childhood adversities in FEP patients when compared to control subjects have been observed in an overlapping sample (Lindgren et al., 2017), and have also been shown to be more prevalent in TRS patients when compared to treatment responsive patients diagnosed with schizophrenia or schizoaffective disorder (Hassan and De Luca, 2015). Interestingly, a lower score for somatic concern and a less active lifestyle seemed to predict future TRS, results which have not to our knowledge previously been observed.

## 5 Strengths and limitations

The HEPS cohort represents a heterogenous FEP patient cohort, where baseline evaluation was performed in the early stages of treatment. Thus, this study sample closely mimics the heterogenous population presenting with psychotic symptoms, who most likely would benefit most from early interventions. In this study we decided to gather TRS information from patient records instead of prescription-based data. Thus, the outcome was verified to maximize accuracy, and at least in this study, prescription-based outcomes would have resulted in two of the seven patients who did not respond to traditional or second-generation antipsychotic medication being misclassified. Conversely, the sample size in this study is small, and the study is most likely underpowered and prone to overfitting (Varoquaux, 2018). In addition, the study was restricted to one scanner, patients were enrolled from a relatively small area in a mostly urban setting, and we did not use an external validation cohort. This is the first study to integrate ISC data when classifying TRS patients, and it should be regarded as a pilot study, supporting the notion that fMRI data can provide valuable information on the development of TRS. The final accuracy of the model was relatively high, most likely not caused by chance as shown by permutation tests. In addition, we believe we have taken all necessary steps to avoid data leakage between training and testing datasets.

## 6 Conclusions

While our results are not applicable to clinical settings, they support the growing body of evidence that TRS could be predicted using pattern recognition models. Due to fear of side effects, clozapine initiation is often delayed, and the decision to initiate treatment is solely based on an adequate follow-up and treatment failure. In such cases, CDSS could aid clinicians as they could lend a prospective insight into the course of the disease. Disorder states such as TRS are most likely complex in nature, and it is unlikely that a single biomarker allowing for infallible accuracy will materialize. Thus, further research should focus on predicting TRS in larger sample sizes, spanning different centers and multiple modalities. Predicting TRS in a clinical setting remains an elusive yet potentially transformative goal. To benefit patients, outcomes of clinical relevance should be studied to dissect the heterogenous clinical diagnoses that most likely group together multiple different sub-diagnoses. Predicting a diagnosis that does not affect treatment options is of little clinical relevance, especially when the criteria for diagnoses such as schizophrenia change and remain vague. Predicting response to pharmacological and other treatment options will bring far more benefit for patients than allocation into current diagnostic categories.

## Funding

This work was funded by the 10.13039/501100006306Sigrid Jusélius Foundation (J.S.), the 10.13039/501100003125Finnish Cultural Foundation (J.S.), the 10.13039/100010135Medical Society of Finland (J.M.P.), the 10.13039/501100002341Academy of Finland (grants #278171 and #323035 to J.S. and #315861 to T.T.R.), the 10.13039/100008723Finnish Medical Foundation (J.M.P. and T.T.R.), state funding for university-level health research (Hospital District of Helsinki and Uusimaa #TYH2013332, #TYH2014228, #TYH2017128 to T.K.), and the European Union's Seventh Framework Programme for project METSY (# 602478 to J.S.).

## CRediT authorship contribution statement

**Tuukka T. Raij:** Writing – review & editing, Supervision, Resources, Project administration, Methodology, Investigation, Funding acquisition, Data curation, Conceptualization. **Tuula Kieseppä:** Writing – review & editing, Resources, Project administration, Investigation, Funding acquisition, Data curation. **Maija Lindgren:** Writing – review & editing, Resources, Project administration, Investigation, Data curation. **Jaana Suvisaari:** Writing – review & editing, Resources, Project administration, Investigation, Funding acquisition, Data curation. **Jussi Alho:** Writing – review & editing, Software. **Athanasios Gotsopoulos:** Writing – review & editing, Software, Methodology, Formal analysis, Data curation, Conceptualization. **Jonatan Mikael Panula:** Writing – original draft, Visualization, Software, Methodology, Investigation, Funding acquisition, Formal analysis, Data curation, Conceptualization.

## Declaration of Competing Interest

The authors declare that they have no known competing financial interests or personal relationships that could have appeared to influence the work reported in this paper.
