# Supplementary material for: Multimodal prediction of the need of clozapine in treatment resistant schizophrenia; a pilot study in first-episode psychosis
Source: Biomark Neuropsychiatry. 2024 Dec;11:None. doi: 10.1016/j.bionps.2024.100102 (PMC11636528; doi:10.1016/j.bionps.2024.100102)
Supplement: Supplementary file 1 — Supplementary material [file mmc1.docx]

Supplement 1

The Helsinki Early Psychosis Study (HEPS) cohort consists of 97 first episode psychosis (FEP) patients. In addition, the cohort contained 62 control subjects (and seven at risk patients who were not considered in this study). Patients participated in the baseline study when the clinical predictor variables and MRI images were obtained. Participants took part in follow-up studies at two months and 12 months following the baseline study. Treatment resistance and a need for clozapine treatment consideration were first assessed based on data from follow-up studies. At the 12-month follow-up, four of the seven patients who would later be classified as treatment resistance had been initiated on clozapine. Based on patient records, a further three patients were considered treatment resistant at 424, 714 and 1424 days after the baseline study.

The prediction model included 81 preselected clinical variables presented in Supplement Figure 1. Significant uncorrected p-values shown in bold. Additional information on variables presented below table.

Supplement Figure 1. Clinical variables used in predicting treatment resistance in first-episode psychosis patients of the HEPS cohort.

|  | Clozapine+ ^a^ N=7 | Clozapine- ^a^ N=31 | Group Difference ^b^ |
| --- | --- | --- | --- |
| 1. Male | 6 (85.7), (0) | 19 (61.3), (0) | p = 0.238 |
| 2. Age | 25.1, 25.2 (22.4-28.0, 5.1), (0) | 26.8, 25.8 (18.3-39.1, 9.9), (0) | p = 0.883 |
| 3. DUP | 1=3, 2=2, 4=2, (0) | 1=14, 2=11, 3=2, 4=3, (1) | p = 0.662 |
| 4. Marital status | 1=1, 5=6, (0) | 1=2, 2=5, 3=2, 5=22, (0) | p = 0.533 |
| 5. Previously married | 1 (14.3), (0) | 11, (35.5), (0) | p = 0.276 |
| 6. Primary school success | 1=2, 2=4, 3=1, (0) | 1=13, 2=15, 3=3, (0) | p = 0.794 |
| 7. Learning disability | 2 (28.6), (0) | 12, (40.0), (1) | p = 0.575 |
| 8. Support teaching | 2 (28.6), (1) | 8 (34.8), (8) | p = 0.947 |
| 9. Bullying | 0=3, 1=3, (1) | 0=18, 1=7, 2=1, (5) | p = 0.524 |
| 10. Years of education | 13.8, 13.5 (11.5-17.0, 4), (0) | 14.7, 13.0 (10.50-22.0, 5.0), (0) | p = 0.740 |
| 11. Level of education | 1=1, 2=5, 3=1, (0) | 1=3, 2=20, 3=8, (0) | p = 0.788 |
| 12. Occupation | 1=2, 3=3, 5=2, (0) | 1=5, 2=4, 3=11, 5=7, 6=2, 8=1, (1) | p = 0.824 |
| 13. Close relative with psychiatric disorder | 5 (71.4), (1) | 17 (54.8), (0) | p = 0.193 |
| 14. Physical condition | 3.0, 3.0 (2-4, 0.5), (1) | 2.8, 3.0 (1-5, 2), (5) | p = 0.751 |
| 15. Amount of exercise | 1.3, 1.0 (1-2, 1), (1) | 2.1, 2.0 (1-4, 1.5), (6) | **p = 0.046** |
| 16. Smoking currently | 3 (42.9), (1) | 5 (16.1), (3) | p = 0.082 |
| 17. Fagerström test for nicotine dependence | 4.3, 5.0 (0-8, 8), (1) | 1.5, 0.0 (0-7, 3), (4) | p = 0.098 |
| 18. Cannabis ever used | 5 (71.4), (0) | 15, (48.4), (0) | p = 0.270 |
| 19. Cannabis amount used | 0=2, 1=1, 2=2, 4=1, (1) | 0=14, 1=7, 2=2, 3=1, 4=3 (4) | p = 0.464 |
| 20. Treatment adhesion | 4.9, 5.0 (3-6, 2), (0) | 5.2, 5.0 (3-7, 1), (0) | p = 0.483 |
| 21. Clinical insight | 15.7, 15.0 (12-20, 5), (0) | 16.1, 17.0 (4-23, 5.0), (2) | p = 0.557 |
| 22. Adversity factor score | 0.5, 0.4 (-0.2-1.5, 0.7), (1) | 0.1, -0.1 (-0.5-1.3, 1.1), (4) | p = 0.109 |
| 23. S-hs-CRP | 4.2, 1.7 (0.2-12.5, 10.0), (0) | 1.0, 0.4 (0.1-4.3, 1.5), (3) | p = 0.066 |
| 24. BMI | 22.4, 22.9 (18.1-31.3, 5.7), (0) | 23.8, 22.7 (18.0-35.8, 3.7), (0) | p = 0.438 |
| 25. MDQ sum | 6.2, 6.0 (3-10, 4.8), (1) | 5.6, 5.0 (0-13, 8), (4) | p = 0.733 |
| 26. Remission at baseline | 0 (0.0), (0) | 6 (19.4), (0) | p = 0.205 |
| 27. Audit | 11.8, 8.5 (2-30, 16), (1) | 7.1, 6.8 (0-21, 12), (4) | p = 0.260 |
| 28. BDI | 9.3, 10.5 (2-16, 10.3), (1) | 12.4, 9.0 (1-44, 13), (4) | p = 0.803 |
| 29. BAI | 18.3, 21.9 (4-32, 17.5), (1) | 13.6, 10.0 (0-42, 12.9), (4) | p = 0.281 |
| 30. OCI-R | 15.0, 17.0 (5-24, 16), (1) | 14.1, 14.0 (0-54, 15.0), (4) | p = 0.569 |
| 31. BCIS self-reflectiveness | 17.5, 18.5 (13-22, 7.5), (1) | 15.7, 16.0 (5-22, 6.5), (6) | p = 0.542 |
| 32. BECK self-certainty | 7.0, 7.5 (4-8, 1.8), (1) | 7.2, 8.0 (1-13, 3.0), (6) | p = 0.751 |
| 33. SOFAS | 37.9, 40.0 (30-40, 5), (0) | 44.3, 40.0 (30-65, 10.0), (0) | p = 0.083 |
| 34. GAF | 32.0, 35.0 (15-40, 3), (0) | 39.7, 40.0 (30-65, 5), (0) | **p = 0.027** |
| SANS scale at admission |  |  |  |
| 35. SANS expressive | 0.6, 0.0 (0-2.5, 1), (0) | 0.4, 0.0 (0-3, 0.5), (0) | p = 0.685 |
| 36. SANS experiential | 2.2, 2.0 (1.5-3.0, 1.5), (0) | 1.6, 1.5 (0-3.5, 2), (0) | p = 0.219 |
| 37. SANS total negative | 1.4, 1.3 (0.8-2.8, 1.3), (0) | 1.0, 1.3 (0-2.8, 1.3), (0) | p = 0.265 |
| 38. SANS alogia | 0.1, 0.0 (0.0-1.0, 0.0), (0) | 0.3, 0.0 (0-3, 0), (0) | p = 0.999 |
| 39. SANS anhedonia | 1.4, 1.0 (0.0-3.0, 1.0), (0) | 1.5, 2.0 (0-4, 3), (0) | p = 0.999 |
| 40. SANS avolition | 3.0, 3.0 (2.0-4.0, 0), (0) | 1.8, 2.0 (0-3, 2), (0) | **p = 0.019** |
| BPRS-E scale at admission |  |  |  |
| 41. BPRS1 somatic concern | 1.4, 1.0 (1-2, 0), (0) | 2.0, 1.0 (1-6, 2), (0) | p = 0.249 |
| 42. BPRS2 anxiety | 3.6, 3.0 (2-6, 1), (0) | 3.3, 4.0 (1-6, 4), (0) | p = 0.768 |
| 43. BPRS3 depression | 2.3, 2.0 (1-5, 3), (0) | 2.6, 2.5 (1-5, 3), (0) | p = 0.740 |
| 44. BPRS4 suicidality | 2.1, 1.0 (1-7, 1), (0) | 1.7, 1.0 (1-5, 2), (0) | p = 0.685 |
| 45. BPRS5 guilt | 2.7, 2.0 (1-7, 2), (0) | 2.3, 2.0 (1-5, 2), (0) | p = 0.941 |
| 46. BPRS6 hostility | 1.1, 1.0 (1-2. 0), (0) | 1.3, 1.0 (1-4, 0), (0) | p = 0.912 |
| 47. BPRS7 elevated mood | 1.0, 1.0 (1-1, 0), (0) | 1.2, 1.0 (1-3, 0), (0) | p = 0.438 |
| 48. BPRS8 grandiosity | 2.3, 1.0 (1-7, 3), (0) | 1.4, 1.0 (1-6, 0), (0) | p = 0.530 |
| 49. BPRS9 suspiciousness | 4.3, 5.0 (1-6, 4), (0) | 3.5, 4.0 (1-7, 4), (0) | p = 0.299 |
| 50. BPRS10 hallucinations | 4.1, 5.0 (1-6, 4), (0) | 2.9, 2.0 (1-6, 4), (0) | p = 0.145 |
| 51. BPRS11 unusual thought content | 5.3, 6.0 (1-7, 1), (0) | 3.7, 4.0 (1-7, 4), (0) | **p = 0.015** |
| 52. BPRS12 bizarre behavior | 1.4, 1.0 (1-2, 0), (0) | 1.5, 1.0 (1-6, 1), (0) | p = 0.606 |
| 53. BPRS13 self-neglect | 1.4, 1.0 (1-3, 1), (0) | 1.3, 1.0 (1-4, 0), (0) | p = 0.797 |
| 54. BPRS14 disorientation | 1.4, 1.0 (1-2, 0), (0) | 1.0, 1.0 (1-1. 0), (0) | p = 0.580 |
| 55. BPRS15 conceptual disorganization | 1.0, 1.0 (1-1, 0), (0) | 1.2, 1.0 (1-3, 0), (0) | p = 0.530 |
| 56. BPRS16 blunted affect | 2.1, 1.0 (1-5, 2), (0) | 1.6, 1.0 (1-4, 1), (0) | p = 0.530 |
| 57. BPRS17 emotional withdrawal | 1.3, 1.0 (1-2, 1), (0) | 1.3, 1.0 (1-4, 0), (0) | p = 0.713 |
| 58. BPRS18 motor retardation | 1.4, 1.0 (1-3, 1), (0) | 1.1, 1.0 (1-3, 0), (0) | p = 0.374 |
| 59. BPRS19 tension | 1.4, 1.0 (1-2, 0), (0) | 1.3, 1.0 (1-4, 0), (0) | p = 0.999 |
| 60. BPRS20 uncooperativeness | 1.4, 1.0 (1-2, 0), (0) | 1.1, 1.0 (1-2, 0), (0) | p = 0.854 |
| 61. BPRS21 excitement | 1.0, 1.0 (1-1, 0), (0) | 1.1, 1.0 (1-3, 0), (0) | p = 0.713 |
| 62. BPRS22 distractibility | 1.0, 1.0 (1-1, 0), (0) | 1.2, 1.0 (1.3, 0), (0) | p = 0.606 |
| 63. BPRS23 motor hyperactivity | 1.0, 1.0 (1-1, 0), (0) | 1.2, 1.0 (1-3, 0), (0) | p = 0.606 |
| 64. BPRS24 mannerism and posturing | 1.0, 1.0 (1-1, 0), (0) | 1.0, 1.0 (1-2, 0), (0) | p = 0.912 |
| 65. BPRS sum | 45.9, 44.0 (33-68, 8), (0) | 41.7, 42.0 (24-65, 12), (0) | p = 0.555 |
| 66. BPRS10+BPRS11+BPRS12+  BPRS15 | 11.6, 13.0 (4-16, 4), (0) | 9.3, 10.0 (4-20, 6), (0) | p = 0.125 |
| 67. BPRS10+BPRS11 | 9.4, 11.0 (2-13, 4), (0) | 6.5, 6.0 (2-13, 6), (0) | **p = 0.040** |
| 68. BPRS10+BPRS11+BPRS15 | 10.4, 12.0, (3-14, 4), (0) | 7.8, 8.0 (3-14, 6), (0) | p = 0.059 |
| 69. BPRS12+BPRS14+BPRS15+  BPRS24 | 5.4, 4.0 (4-8, 4), (0) | 5.4, 5.0 (4-11, 2), (0) | p = 0.912 |
| 70. BPRS16+BPRS17+BPRS18 | 4.9, 3.0 (3-10, 3), (0) | 4.0, 3.0 (3-10, 1), (0) | p = 0.580 |
| 71. BPRS16+SANS1-3 | 6.7, 6.0 (4-12, 5), (0) | 5.1, 6.0 (1-12, 5), (0) | p = 0.265 |
| BPRS-E scale worst symptoms before admission |  |  |  |
| 72. BPRS1 somatic concern | 1.9, 1.0 (1-6, 1), (0) | 2.6, 1.0 (1-7, 3), (1) | p = 0.435 |
| 73. BPRS5 guilt | 2.7, 2.0 (1-7, 2), (0) | 2.7, 3.0 (1-7, 2), (0) | p = 0.658 |
| 74. BPRS8 grandiosity | 2.6, 2.4 (1-7, 5), (0) | 2.3, 1.0 (1-7, 1), (0) | p = 0.971 |
| 75. BPRS9 suspiciousness | 4.7, 6.0 (1-7, 5), (0) | 5.0, 6.0 (1-7, 4), (0) | p = 0.685 |
| 76. BPRS10 hallucinations | 5.9, 6.0 (2-7, 1), (0) | 4.7, 6.0 (1-7, 5), (0) | p = 0.205 |
| 77. BPRS11 unusual thought content | 6.5, 6.5 (6-7, 1), (1) | 6.4, 7.0 (3-7, 1), (0) | p = 0.740 |
| 78. BPRS12 bizarre behavior | 3.1, 3.0 (1-6, 5), (0) | 3.7, 4.0 (1-6, 4), (0) | p = 0.580 |
| 79. BPRS13 self-neglect | 2.0, 1.0 (1-4, 2), (0) | 2.0, 1.0 (1-5, 2), (0) | p = 0.912 |
| 80. BPRS10+BPRS11+BPRS12+  BPRS15 | 15.8, 15.0 (14-18, 3.25), (1) | 16.1, 16.0 (9-21, 5.0), (0) | p = 0.580 |
| 81. BPRS10+BPRS11 | 12.2, 13.0 (8-14, 2.25), (1) | 11.2, 12.0 (6-14, 6), (0) | p = 0.587 |

^a^ Frequency (%) or mean, median (range, IQR), (missing values N)

^b^ Mann-Whitney U-test or Pearson Chi-square test. Significant uncorrected p-values shown in bold.

3. Duration of untreated psychosis (DUP)
 1 = < 1 month
 2 = 1 – 6 months
 3 = 6 – 12 months
 4 = > 12 months

4. Marital status

1 = married

2 = cohabitation

3 = divorced

4 = widow

5 = unmarried

6. Primary school success: How well do you think you performed in primary school when compared to your peers?

1 = better than average

2 = average

3 = worse than average

7. Did you experience learning difficulties lasting more than one semester?

1 = yes

0 = no

8. Did you receive supportive education for these difficulties?

1 = yes

0 = no

9. Were you bullied in school

0 = no

1 = yes

2 = cannot say

11. Level of education

1 = basic

2 = secondary

3 = high

12. Occupation

1 = working full-time

2 = working part-time

3 = student

4 = receiving disability pension or cash rehabilitation benefit

5 = unemployed

6 = family manager/family caregiving

7 = military service/civilian service

8 = other

14. How would you rate your physical condition?

1 = good

2= fairish

3 = average

4 = below average

5 = poor

15. How much do you exercise?

1 = “In my spare time I read, watch TV and do chores where I don't move a lot and that don't burden me physically”

2 = “In my spare time, I walk, bike and move in other ways at least 4 hours a week”

3 = “In my spare time, I do physical exercise on average at least 3 hours a week”

4 = “In my spare time, I train regularly in a competitive sport multiple times a week (also answer this if you train full-time for competitive sports)”

19. During the past 12 months, how many times have you used cannabis

0 = not at all

1 = 1-5 times

2 = 6-10 times

3 = 11-50 times

4 = over 50 times

20. Treatment adhesion based on (David et al., 1992)

21. Clinical insight based on (David et al., 1992)

22. Adversity factor score containing combined results of 10 questions examining adverse events in childhood. (Lindgren et al., 2017)

23. High-sensitivity C-reactive protein

24. Body mass index

25. Mood Disorder Questionnaire (Hirschfeld et al., 2000)

26. Remission at baseline. Defined as BPRS-E items 10+11+15+16+24 < 4 and SANS alogia + anhedonia + avolition < 3 (Andreasen, 1989; Ventura et al., 1993)

27. Alcohol Use Disorders Identification Test. (Saunders et al., 1993)

28. Beck Depression Inventory (Beck et al., 1961)

29. Beck Anxiety Inventory (Beck et al., 1988)

30. Obsessive-Compulsive Inventory (Foa et al., 2002)

31-32 Beck Cognitive Insight Scale (Beck et al., 2004)

33. Social and Occupational Functioning Assessment Scale (Goldman et al., 1992)

34. Global Assessment of Functioning Scale (Lehman, 1983)

35.-40. The Scale for the Assessment of Negative Symptoms (SANS) (Andreasen, 1989)

41.-71. Expanded Brief Psychiatric Rating Scale as evaluated at admission (Ventura et al., 1993)

72.-81. Expanded Brief Psychiatric Rating Scale. Estimated most severe symptoms before admission.

**References**

Andreasen, N. C. (1989). The Scale for the Assessment of Negative Symptoms (SANS): conceptual and theoretical foundations. *Br J Psychiatry Suppl*(7), 49-58. <https://www.ncbi.nlm.nih.gov/pubmed/2695141>

Beck, A. T., Baruch, E., Balter, J. M., Steer, R. A., & Warman, D. M. (2004). A new instrument for measuring insight: the Beck Cognitive Insight Scale. *Schizophr Res*, *68*(2-3), 319-329. <https://doi.org/10.1016/S0920-9964(03)00189-0>

Beck, A. T., Epstein, N., Brown, G., & Steer, R. A. (1988). An inventory for measuring clinical anxiety: psychometric properties. *J Consult Clin Psychol*, *56*(6), 893-897. <https://doi.org/10.1037//0022-006x.56.6.893>

Beck, A. T., Ward, C. H., Mendelson, M., Mock, J., & Erbaugh, J. (1961). An inventory for measuring depression. *Arch Gen Psychiatry*, *4*, 561-571. <https://doi.org/10.1001/archpsyc.1961.01710120031004>

David, A., Buchanan, A., Reed, A., & Almeida, O. (1992). The assessment of insight in psychosis. *Br J Psychiatry*, *161*, 599-602. <https://doi.org/10.1192/bjp.161.5.599>

Foa, E. B., Huppert, J. D., Leiberg, S., Langner, R., Kichic, R., Hajcak, G., & Salkovskis, P. M. (2002). The Obsessive-Compulsive Inventory: development and validation of a short version. *Psychol Assess*, *14*(4), 485-496. <https://www.ncbi.nlm.nih.gov/pubmed/12501574>

Goldman, H. H., Skodol, A. E., & Lave, T. R. (1992). Revising axis V for DSM-IV: a review of measures of social functioning. *Am J Psychiatry*, *149*(9), 1148-1156. <https://doi.org/10.1176/ajp.149.9.1148>

Hirschfeld, R. M., Williams, J. B., Spitzer, R. L., Calabrese, J. R., Flynn, L., Keck, P. E., Jr., Lewis, L., McElroy, S. L., Post, R. M., Rapport, D. J., Russell, J. M., Sachs, G. S., & Zajecka, J. (2000). Development and validation of a screening instrument for bipolar spectrum disorder: the Mood Disorder Questionnaire. *Am J Psychiatry*, *157*(11), 1873-1875. <https://doi.org/10.1176/appi.ajp.157.11.1873>

Lehman, A. F. (1983). The effects of psychiatric symptoms on quality of life assessments among the chronic mentally ill. *Evaluation and Program Planning*, *6*(2), 143-151. <https://doi.org/https://doi.org/10.1016/0149-7189(83)90028-9>

Lindgren, M., Mantyla, T., Rikandi, E., Torniainen-Holm, M., Morales-Munoz, I., Kieseppa, T., Mantere, O., & Suvisaari, J. (2017). Childhood adversities and clinical symptomatology in first-episode psychosis. *Psychiatry Res*, *258*, 374-381. <https://doi.org/10.1016/j.psychres.2017.08.070>

Saunders, J. B., Aasland, O. G., Babor, T. F., de la Fuente, J. R., & Grant, M. (1993). Development of the Alcohol Use Disorders Identification Test (AUDIT): WHO Collaborative Project on Early Detection of Persons with Harmful Alcohol Consumption--II. *Addiction*, *88*(6), 791-804. <https://doi.org/10.1111/j.1360-0443.1993.tb02093.x>

Ventura, J., Lukoff, D., Nuechterlein, K., Liberman, R., Green, M., Shaner, A., Green, M. E., Shaner, D., & Lieberman, R. (1993). Brief Psychiatric Rating Scale (BPRS) Expanded Version (4.0): Scales, Anchor Points, and Administration Manual.
